# Multifaceted Role of Nanomaterials in Modulating In Vitro Seed Germination, Plant Morphogenesis, Metabolism and Genetic Engineering

**DOI:** 10.3390/plants12173126

**Published:** 2023-08-30

**Authors:** Ashutosh Pathak, Shamshadul Haq, Neelam Meena, Pratibha Dwivedi, Shanker Lal Kothari, Sumita Kachhwaha

**Affiliations:** 1 Department of Botany, University of Rajasthan, Jaipur 302004, Rajasthan, India; ashutosh.pathak87@gmail.com (A.P.); shamshadbiotech@gmail.com (S.H.); meenaneelam2702@gmail.com (N.M.); dwivedi.pr2269@gmail.com (P.D.); 2 Amity Institute of Biotechnology, Amity University Rajasthan, Jaipur 303002, Rajasthan, India; slkothari28@gmail.com

**Keywords:** crops, genetic engineering, in vitro cultures, nanoparticles, regeneration, seedlings, secondary metabolites

## Abstract

The agricultural practices of breeding, farm management and cultivation have improved production, to a great extent, in order to meet the food demands of a growing population. However, the newer challenges of climate change, global warming, and nutritional quality improvement will have to be addressed under a new scenario. Plant biotechnology has emerged as a reliable tool for enhancing crop yields by protecting plants against insect pests and metabolic engineering through the addition of new genes and, to some extent, nutritional quality improvement. Plant tissue culture techniques have provided ways for the accelerated clonal multiplication of selected varieties with the enhanced production of value-added plant products to increase modern agriculture. The in vitro propagation method has appeared as a pre-eminent approach for the escalated production of healthy plants in relatively shorter durations, also circumventing seasonal effects. However, there are various kinds of factors that directly or indirectly affect the efficiency of in vitro regeneration like the concentration and combination of growth regulators, variety/genotype of the mother plant, explant type, age of seedlings and other nutritional factors, and elicitors. Nanotechnology as one of the latest and most advanced approaches in the material sciences, and can be considered to be very promising for the improvement of crop production. Nanomaterials have various kinds of properties because of their small size, such as an enhanced contact surface area, increased reactivity, stability, chemical composition, etc., which can be employed in plant sciences to alter the potential and performance of plants to improve tissue culture practices. Implementing nanomaterials with in vitro production procedures has been demonstrated to increase the shoot multiplication potential, stress adaptation and yield of plant-based products. However, nanotoxicity and biosafety issues are limitations, but there is evidence that implies the promotion and further exploration of nanoparticles in agriculture production. The incorporation of properly designed nanoparticles with tissue culture programs in a controlled manner can be assumed as a new pathway for sustainable agriculture development. The present review enlists different studies in which treatment with various nanoparticles influenced the growth and biochemical responses of seed germination, as well as the in vitro morphogenesis of many crop species. In addition, many studies suggest that nanoparticles can be useful as elicitors for elevating levels of important secondary metabolites in in vitro cultures. Recent advancements in this field also depict the suitability of nanoparticles as a promising carrier for gene transfer, which show better efficiency than traditional *Agrobacterium*-mediated delivery. This review comprehensively highlights different in vitro studies that will aid in identifying research gaps and provide future directions for unexplored areas of research in important crop species.

## 1. Introduction

Owing to their minuscule size, NPs acquire novel and unique properties that differ from their bulkier counterparts [1], giving rise to breakthrough technology with application-based solutions in many sectors of agriculture and plant biotechnology. However, the release of NPs into the environment has raised concern because of their toxic effects on the environment and human health [2]. Moreover, the release of NPs into the environment could result in their entry and accumulation in agricultural soils from bio-solids impregnated with NPs through the application of sewage sludge for agricultural purposes [3]. Thus, the application of NPs in plant tissue cultures is promising, as this technique is used to screen different aspects of plants’ growth and development, as well as to engage in genetic manipulation, bioactive compound production and plant improvement [4]. It has been noted that NPs have a positive impact because of their reduced size, elevated reactivity, mass-to-area ratio and other physico-chemical properties, but the negative effects of NPs have also been noted, which mainly depend on the type of metal, dissolution power and plant species [5,6]. In recent years, the application of NPs has demonstrated a positive effect on callus induction, organogenesis, somatic embryogenesis, genetic transformation and secondary metabolite production. Although there are a number of reviews on the application of nanomaterials in plants [7,8,9,10] and in agriculture [11,12] to mitigate various stresses [13,14], reviews on their application in plant tissue culture are scant [15]. Thus, this review comprises numerous studies that were conducted to explore the in vitro application of NPs on commercially important crops with respect to various aspects like preventing contamination, impact on seed germination, production of metabolites, induction of morphogenesis, biochemical and molecular changes, and genetic engineering.

## 2. Efficiency of NPs in Eliminating Contamination

The production of healthy plantlets is a prime concern behind the technique of plant tissue culture but microbial contamination is a common problem faced during this procedure. Conventionally, antibiotics are employed to eliminate microbes, but their frequent application can negatively affect plant tissue growth, e.g., antibiotics like carbenicillin and cefotaxime inhibit plant cell growth, organogenesis and embryogenesis [16,17]. Reports suggest that streptomycin and chloramphenicol interact with protein synthesis, rifampicin hinders nucleic acid synthesis and penicillin inhibits cell-wall membrane synthesis [18,19]. There is also the risk of a decreased genetic stability and lower regeneration capability of plants when a high level of antibiotics is used [20]. Nanomaterials are an alternative because of their distinctive features, which have been shown to possess antifungal and antibacterial properties that restrict microbial growth in in vitro cultures resulting in the successful mass propagation of selected species [21]. Silver nanoparticles (AgNPs) have been considered one of the better options, as the anchoring and penetration of Ag ions into microbes alter the cellular signals, via dephosphorylation, of key peptide substrates on tyrosine [22,23]. Another study suggested that Ag^+^ ions cause a reduction in DNA replication, as well as inactivate the thiol group in proteins, that ultimately reduces microbial growth [24]. Similarly, Min et al. [25] reported that AgNPs restrict the growth of sclerotium-forming phytopathogenic fungi and, hence, can become an alternative to pesticides. AgNPs have been employed to reduce contamination during in vitro cultures of *Olea europaea* L. [26], *Nicotiana tabacum* L. [27,28], *Gerbera jamesonii* Bolus ex Hook.f. [29], *Solanum tuberosum* L. [21], almond x peach (G x N15) hybrid rootstock [30], *Rosa hybrida* L. [31], *Vitis vinifera* L. [32], *Vanilla planifolia* Jacks. ex Andrews [33], and *Phoenix dactylifera* L. cv. Sewi and Medjool [34]. In addition, combined treatment with nanosilver and nano-iron particles was reported to have a significant effect on decreasing the contamination rate in *Fragaria* × *ananassa* L. cv. Roby Gem [35]. Similarly, biosynthesized silver, chitosan, and selenium NPs were tested for their antimicrobial potential for the in vitro multiplication of three olive cultivars (Koroneiki, Picual, and Manzanillo). Of all the three NPs, AgNPs showed the best antimicrobial properties in all cultivars [36]. However, some studies have also suggested that the concentrations of AgNPs played a crucial role in culture growth as higher concentrations might induce adverse effects on explant response [3,21]. The phytotoxic effect of higher AgNPs has been observed in crop plants of *Phaseolus radiatus* L. and *Sorghum bicolor* (L.) Moench [37]. Whereas in tomato and potato plants, it has been reported that lower concentrations of AgNPs with longer exposure time effectively reduced the contamination without hampering explant viability [38].

Titanium dioxide (TiO_2_) is another NP that has gained attention due to its antimicrobial potential, as it has photocatalytic properties to eliminate contamination from various sources, but its toxicity against microbial growth depends on the intensity and wavelength of light with concentration and particle size [19]. TiO_2_ reacts with water molecules and forms free radicals like OH, HO_2_, and H_2_O_2_ which in turn results in the oxidation of bacterial cells, suggesting that the photo-activation of TiO_2_ via UV irradiation retards the bacterial growth [39,40]. It has been evaluated that the addition of TiO_2_NPs in the Murashige and Skoog (MS) [41] medium enhanced the microbial resistance during the micropropagation of tobacco [27], *S. tuberosum* [19], and *Hordeum vulgare* L. [42]. Zinc oxide nanoparticles (ZnONPs) have eliminated nine strains of bacteria (*Bacillus megaterium*, *Cellulomonas uda*, *C. flarigena*, *Corynebacterium panrometabolum*, *Erwinia cypripedii*, *Klebsiella* spp., *Pseudomonas* spp., *Proteus* spp., and *Staphylococcus* spp.) and four fungal species (*Aspergillus* spp., *Candida* spp., *Fusarium* spp., and *Penicillium* spp.) which increased difficulties during banana micropropagation [43]. Thus, it can be observed that although nanomaterials at higher concentrations have been proven as toxic for plant growth, they can be employed as disinfectant agents for the in vitro multiplication of various economically important crops. The majority of the reports used Ag, TiO_2_, and Zn-based NPs for the inhibition of microbial growth during in vitro propagation, but new types of NPs should also be assessed. In this regard, various kinds of advanced nanomaterials like graphene, graphite, quantum dots, carbon nanotubes, polymer dendrimers, and atomic clusters will provide enough scope for the study; along with this, evaluations of concentrations, sizes, and types of NPs on various crop species and type of explant are also needed [44].

## 3. Influence of NPs on Seed Germination

Seed germination is a crucial stage for crop development since young seedlings are more vulnerable to biotic and abiotic stresses [45]. Therefore, lots of efforts to improve the efficiency of seed germination are published from time to time with new technological interventions. Studies to analyze the effect of NPs have been conducted during the last few years, and it was observed that genotype, variety, seed age, and environmental conditions determined the response to NPs [46]. Yasur and Rani [47] and Hatami [48] suggested that the water uptake during seed germination is critical because seeds are relatively dry and requires a substantial amount of water to initiate cellular metabolism and growth. The positive effects of NPs on germination begin with the high capability of NPs to penetrate the seed coat and promote water uptake along with the absorption of nutrients in the seed [49]. Mehrian et al. [50] documented that NP treatment accelerated seed germination from better water uptake by the seeds during the initial days, whereas a decrease in germination efficiency was noted as time passed because of the breakdown of stored nutrients or alternations in permeability properties of the cell membrane. Similarly, Rizwan et al. [51] noted that NPs can penetrate through the seed coat and affect the development processes of embryos through stimulation of the enzymes of metabolic processes. During the radicle appearance stage of seed germination, root apex tissues come in contact with NPs, which then move into the rhizodermis through the apoplast with endocytosis. In the root, they flow towards the plant secretory tissue using symplastic pathways and translocate to other plant organs. However, it has been noted that NPs at a high concentration result in a perforation of the cell wall and penetrate the protoplast and damage the root cell vacuoles. This triggers more production of reactive oxygenspecies (ROS) and it causes a blockage of electron transfer which induces oxidative stress. NPs also up-regulate the genes involved in cell division and carbon/nitrogen metabolism, and the negative effects observed in seedling growth are due to chromosomal aberrations and mitotic abnormalities. This leads to a decrease in cell division of the root meristem, hormonal imbalance, ROS over-production, and increased levels of lipid peroxidation [52]. The increased oxidative stress, in turn, increases hydrogen peroxide (H_2_O_2_) contents, activities of malondialdehyde (MDA), catalases (CAT), peroxidases (POD), and superoxide dismutase (SOD), as well as the production of compounds having antioxidant activities like phenolics and flavonoids [53]. Many studies have documented that NPs exert positive or negative influences on seed germination, seedling biomass as well as biochemical and metabolite contents. In the present review, we have taken only those examples where NPs were added into the media and not where seeds were placed on filter paper or water agar media after sonication treatment with NPs.

### 3.1. AgNPs

In the majority of the studies, NPs’ effect has been evaluated under in vivo conditions [54], but few were tested under in vitro conditions on the culture media. It is also observed that most reports suggested the usage of AgNPs (Table 1), e.g., Lee et al. [37] recorded a negative effect of AgNPs on *P. radiates* and *S. bicolour* seedling growth. Similarly, the growth of *Physalis peruviana* L. seedlings also decreased along with chlorophyll content, but biomass in terms of fresh (FW) and dry weights (DW) was increased. It was also revealed that the seedling growths were not much affected in soil as compared to the agar-based medium. This might be due to changes in the physico-chemical properties of NPs in the soil, as pore water harbours a range of electrolytes that increase the aggregation of AgNPs in soil. These aggregates were larger than the pore size of plant root cells and thus failed to pass through the cells. Greater aggregation may be the principal reason for the reduced phytotoxicity of AgNPs in soil. Thus, the relative germination index is extensively used as an indicator of phytotoxicity, and root growth is one of the sensitive biomarkers for the phytotoxicity assay [55]. Zaka et al. [56] compared AgNPs, gold nanoparticles (AuNPs), and copper nanoparticles (CuNPs) for *Eruca sativa* Mill. and observed that AgNPs increased seed germination, shoot and root lengths, and seed vigour index, whereas the other two adversely affected these parameters (Table 1). Further evaluation unveiled that all the NPs affected the biochemical milieu of the plants differently (Table 2). In another study, green synthesized AgNPs using *Curculigo orchioides* Gaertn. were found to exert a positive influence on seedling growth and biomass of *Oryza sativa* L. cv. Swarna. When the germinated seedlings were biochemically analyzed, an increase in chlorophyll, flavonol contents and enzymes (POD, SOD, CAT, APX, and GR) activities, and a decrease in phenolics, flavonoids, H_2_O_2_, and MDA contents were observed. The gene expression analyses revealed that the SOD gene was down-regulated, whereas genes for CAT and ascorbate peroxidase (APX) were up-regulated after AgNP treatment [57]. Similarly, increased seed germination, seed vigour index, shoot and root lengths, and fresh and dry biomass in *Pennisetum glaucum* (L.) R. Br. after the addition of AgNPs in the medium was reported [58]. The maximum germination was recorded at 40 ppm; at this concentration of AgNPs, mild activities of 2,2-Diphenyl-1-picrylhydrazyl (DPPH), SOD activities and proline content were recorded that significantly increased at higher dose of AgNPs. On the contrary, phenolic contents were higher at optimum germination concentration (40 ppm) and lower at higher concentration, whereas flavonoids were lower at 40 ppm and increased at high levels. AgNPs positively influenced the germination and seedling traits of *Brassica oleracea* L. var. *sabellica* ‘Nero di Toscana’ and *Raphanus sativus* L. var. *sativus* ‘Ramona’, whereas these traits were decreased in *Solanum lycopersicum* L. ‘Poranek’. One of the reasons behind decreased growth *S. lycopersicum* might be due to the presence of AgNPs in plasmodesmata, precluding the transport of nutrients that led to a reduction in plant biomass [59]. Recently, Tomaszewska-Sowa et al. [60] observed the effect of AgNPs and AuNPs on *Brassica napus* L., and revealed that application of both NPs decreased shoot and root lengths of seedlings irrespective of treatment time. However, total chlorophylls, carotenoids, anthocyanins, free sugars, and H_2_O_2_ contents were higher, but no major change in phenolics was found. The seed germination of *N. tabacum* was carried out using CTAB- and PVP-coated AgNPs, and coating with CTAB showed a positive influence whereas coating with PVP failed to show any positive effect on germination rate and biomass [61]. Similarly, positive influences of AgNPs have been also documented in *Brassica juncea* (L.) Czern. var. pusajaikisan [62], *Hylocereus undatus* (Haw.) Britton and Rose [63], and *P. vulgaris* [64] (Table 1).

### 3.2. Other Metal and Metal Oxide NPs

Apart from AgNPs, other metal NPs are also used for seedling germination under in vitro conditions; Dehkourdi and Mosavi [69] utilized TiO_2_NPs and documented a positive influence on seed germination as well as on chlorophyll synthesis in *Petroselinum crispum* (Mill.) Fuss, whereas Nair et al. [71] observed that the application of copper oxide nanoparticles (CuONPs) on *Vigna radiata* L. decreased seedling growth in terms of length and biomass. They have also reported that CuONPs decreased chlorophyll and increased proline contents, whereas it increased H_2_O_2_ and MDA contents in the root; however, no change in carotenoid, H_2_O_2_, and MDA contents in the shoot and increased lignification of root cells were detected (Table 2). The negative effect of CuONPs on seedlings of *Cicer arietinum* L. was also documented where decreased growth and biomass have been recorded at all the tried concentrations (50–500 mg/L), and elevated H_2_O_2_ generation, MDA level, and POD activity along with increased lignifications in roots were observed. Further expression analysis revealed that *CuZn-SOD*, *CAT*, and *APX* genes were up-regulated in roots but no change was found in shoots [66]. Similarly, CuONPs, when used for the treatment of *Brassica nigra* (L.) K. Koch, delayed the germination of seedlings and decreased plantlet length and biomass significantly [65]. ZnONPs in the media containing seeds of the same plant negatively influenced seedling growth, shoot FW, and reduced stem diameter as the NP amount increased in the media. However, the treatment increased free radical scavenging activity, total antioxidant capacity, total reducing power, phenolics, and flavonoid contents in the shoot and root of the seedling (Table 2) [65]. Moreover, in seeds of *Linum usitatissimum* L. cv. Barbara, different concentrations of ZnONPs (1, 10, 100, 500, and 1000 mg/L) were tried, and 100 mg/L concentrations proved beneficial in terms of shoot and root lengths as well as seedling biomass, further higher concentrations adversely affected seedling growth [68]. In another study, treatment with multi-walled carbon nanotubes (MWCNTs) showed a positive influence on germination, seedling lengths, as well as biomass in *Glycine max* (L.) Merr. hybrid S42-T4, *H. vulgare* hybrid Robust, and *Zea mays* L. hybrid N79Z 300GT [67]. Unlike the spherical shapes of other NPs, MWCNTs are the allotropes of carbon that are arranged in an elongated, tubular manner with many rolled sheets. Its unique features like functional group, diameter, length, and solubility make its penetration inside the seed coat convenient and it is efficiently translocated in plants [72]. Similar observations have been well documented previously where MWCNTs improve germination, plant growth, and agronomic traits by penetration, and increasing the water and nutrient uptake [73,74].

**Table 2:** Biochemical changes in seedlings and cultures after NP treatment.

Plant	Nanoparticle (NP) Treatment and Culture Type	Biochemical Changes	Reference
*Brassica juncea* var. pusa jaikisan	AgNPs, shoots	Increased chlorophyll and decreased MDA, H_2_O_2_, and proline content, increased CAT, GPX, and APX activities	[62]
*Brassica napus*	AgNPs/AuNPs, shoots	Increased chlorophylls, carotenoids, anthocyanins, free sugars, H_2_O_2_ contents, no change in phenolic content	[60]
*Brassica nigra*	ZnONPs, shoots and roots (seedling), callus	Increased free radical scavenging activity, total antioxidant capacity, total reducing power, phenolic, and flavonoid contents	[65]
*Brassica nigra*	CuONPs, seedling and roots (from leaf and stem derived callus)	Seedlings increased free radical scavenging activity, total phenolic, and flavonoid content, decreased total antioxidant and reducing potential; Roots increased free radical scavenging activity, total antioxidant and reducing potential, total phenolic, and flavonoid contents	[5]
*Brassica oleracea* var. *sabellica* ‘Nero di Toscana’	AgNPs, leaves	Decreased chlorophyll, carotenoid, and anthocyanin contents, no change in phenolic, protein contents and SOD activities, increased GPOX activity	[59]
*Campomanesia rufa*	AgNPs, shoots	No significant difference in SOD activity	[75]
*Caralluma tuberculata*	AgNPs, callus	Increased PAL and free radical scavenging, SOD, POD, CAT, APX activities, total phenolics, and flavonoid contents	[76]
*Cicer arietinum*	CuONPs, seedling	Increased H_2_O_2_ generation, MDA content, POD activity, and lignification in roots	[66]
*Cichorium intybus*	Fe_2_O_3_NPs, hairy roots	Increased hairy root growth, total phenolic, and flavonoid contents	[77]
*Corylus avellana* cv. Gerd Eshkevar	AgNPs, cell suspension	Increased CAT, APX, H_2_O_2_, PAL activities, decreased SOD and POD activities, and total soluble phenol content	[78]
*Corylus avellana* cv. Gerd Eshkevar	AgNPs, cell suspension	Increased MDA, total phenolic, anthocyanin, and flavonoid contents	[79]
*Cucumis anguria*	AgNPs, hairy roots	Increased total phenolic and flavonoid contents, and antioxidant activities	[80]
*Eruca sativa*	AuNPs, CuNPs, and AgNPs, seedling	AuNPs decreased total antioxidant capacity, total phenolic and flavonoid contents, increased DPPH, SOD and POD activities, no change in protein content; CuNPs decreased total antioxidant capacity, DPPH activity, protein content, increased total phenolic, and flavonoid contents, SOD and POD activities; AgNP decreased total antioxidant capacity, DPPH activity, decreased total phenolics and flavonoid contents, POD activity, increased SOD activity, no change in protein	[56]
*Fragaria* × *ananassa* cv. Queen Elisa	FeNPs, shoots	Increased chlorophyll a, chlorophyll b, total chlorophyll, carotenoid, total carbohydrates, total protein, and total free proline and iron contents, decreased H_2_O_2_ and MDA content, higher SOD and POD activities	[81]
*Linum usitatissimum* cv. Kerman Shahdad	ZnONPs/TiO_2_NPs, cell suspension	Increased PAL and CAD activities, and total phenol content	[82]
*Linum usitatissimum* cv. Barbara	ZnONPs, seedling and callus	Increased ROS production, membrane lipid peroxidation, protein carbonylation and 8-oxo guanine formation, SOD, POD, radical scavenging activities, total phenolics, and flavonoid contents	[68]
*Maerua oblongifolia*	AgNPs, shoots	Higher chlorophyll, total protein and proline contents, and increased activities of antioxidant enzymes	[83]
*Momordica charantia*	AgNPs, cell suspension	Increased MDA, H_2_O_2_, total phenolics and flavonoid contents, and antioxidant activity	[84]
*Musa paradisiacal* cv. Grand Nain	ZnNPs and ZnONPs, shoots	Higher proline, chlorophyll, and antioxidant enzymes activities	[43]
*Musa* spp.	AgNPs, shoots	Increased chlorophyll content	[85]
*Nicotiana benthamiana*	CH-ZnO, callus	Increased chlorophyll, carotenoid, proline contents and PAL and AO activities, decreased MDA and H_2_O_2_ levels	[86]
*Nicotiana tabacum* cv. Bright Yellow-2	ZnONPs, cell suspension	Decreased dehydrogenase, oxidoreductase SOD, POD and APX activities, increased GR, PAL, protease, caspase-like and acid phosphatases activities, and total phenolic content	[87]
*Oryza sativa* cv. Swarna	AgNPs, seedling leaves	Increased chlorophyll and flavonol contents and POD, SOD, CAT, APX and GR activities, decreased phenolics, flavonoids, H_2_O_2_ and MDA contents	[57]
*Oryza sativa* cv. IR64	AgNPs, shoot	Decreased MDA, proline and H_2_O_2_ levels	[88]
*Pennisetum glaucum*	AgNPs, seedling	Increased DPPH, proline, SOD, POD, and CAT activities, total phenolics and flavonoid contents	[58]
*Phoenix dactylifera*	MWCNTs, shoots	Increased flavonoid, chlorophylls and carotenoid, nutrient contents, decreased phenolics and tannin contents, SOD, GPOX, and GR activities	[89]
*Phoenix dactylifera* cv. Hayani	AgNPs, somatic embryos	Increased chlorophyll content	[90]
*Physalis peruviana*	AgNPs, seedling derived shoots and shoots	Seedling derived shoots- increased CAT and APX activity, and decreased chlorophyll content, SOD and MDA activities;Shoots- no change in SOD, APX and MDA levels, decreased CAT activity	[70]
*Raphanus sativus* var. sativus ‘Ramona’	AgNPs, leaves	Increased carotenoid, phenolic contents, and SOD activity, decreased chlorophyll, anthocyanins, protein contents, and GPOX activity	[59]
*Saccharum* spp. cv. Mex 69-290	AgNPs, leaves	Increased N, Ca, Mg, Fe, Cu, Zn, Mn, and decreased P, K, and B content, higher total phenolics, ROS and lipid peroxidation contents, and antioxidant activity	[91]
*Simmondsia chinensis*	MWCNTs, shoots	Increased total tannin content and antioxidant activities, decreased phenolics and flavonoid contents	[92]
*Solanum lycopersicon*	Fe_3_O_4_NPs, shoots	Increased proline content and osmotic potential	[93]
*Solanum lycopersicum*	ZnONPs, callus	Increased Na, N, P, K, and Zn ionic, protein contents, SOD and GPX activity	[4]
*Solanum lycopersicum* var. Poranek	AgNPs, leaves	Increased chlorophyll, anthocyanins, phenolics, protein contents and SOD and GPOX activities, decreased carotenoid content	[59]
*Solanum tuberosum*	SiO_2_NPs, leaves	Increased antioxidant enzymes activity and expression of proteins	[94]
*Solanum tuberosum* cv. White Desiree	AgNPs, shoots	Increased total chlorophyll, carotenoids, proline, total flavonoids, phenolics, lipid peroxidation and H_2_O_2_ contents, decreased anthocyanins	[95]
*Vanilla planifolia*	AgNPs, shoots	Higher chlorophyll, increased elements like N and B, no change in P, Ca and Mg, and decreased K, Fe, Cu, Zn, Mn, and B contents, higher total phenolics, ROS and lipid peroxidation contents, and antioxidant activity	[33]
*Vigna radiata*	CuONPs, seedling	Decreased chlorophyll and increased proline contents, H_2_O_2_ and MDA contents in root, no change in carotenoid, H_2_O_2_ and MDA contents in shoots	[71]

AgNPs: silver nanoparticles; APX: ascorbate peroxidase; AO: ascorbate oxidase; AuNPs: gold nanoparticles; CAT: catalase; CH-ZnO: chitosan-zinc oxide nano-bioformulation; CuNPs: copper nanoparticles; CuONPs: copper oxide nanoparticles; DPPH: 2,2-diphenyl-1-picrylhydrazyl, FeNPs: iron nanoparticles; Fe_2_O_3_NPs/Fe_3_O_4_NPs: iron oxide nanoparticles; GPX: guaiacol peroxidase; GPOX: glutathione peroxidase; GR: glutathione reductase; H_2_O_2_: hydrogen peroxide; MDA: melondialdehyde; MWCNTs: multi-walled carbon nanotubes; PAL: phenylalanine ammonia lyase; POD: peroxidase; ROS: reactive oxygen species; SiO_2_NPs: silicon dioxide nanoparticles; SOD: superoxide dismutase; TiO_2_NPs: titanium dioxide nanoparticles; ZnNPs: zinc nanoparticles; ZnONPs: zinc oxide nanoparticles.

## 4. Modulation of In Vitro Morphogenesis by NPs

The ions supplemented into the culture medium are transported via phloem cells [96] and the apoplastic pathway [97]. In a similar manner, as NPs have extremely small sizes, they enter the explants in a similar way, but their effect mainly depends on NP type, concentration, exposure time, and plant species [98]. Many studies have reported the influence of different types of NPs on the in vitro morphogenesis of various crops which are listed in Table 3.

### 4.1. Metal NPs

In a study on in vitro cultures of *L. usitatissimum*, BAP was coated with different nanoparticles (AuNPs and AgNPs), and AuNPs proved better than AgNPs for callus formation and somatic embryogenesis [99]. On the contrary, the positive effect of AgNPs on the in vitro regeneration of *S. tuberosum* cv. White Desiree has been documented where most of the morphological traits showed improvement (Table 3) along with contents of total chlorophyll, carotenoids, proline, total flavonoids, phenolics, lipid peroxidation, and H_2_O_2_, whereas anthocyanin content was decreased (Table 2). They have concluded that this beneficial effect is possibly due to the inhibition of ethylene perception by AgNPs [95]. In vitro cultures are known to produce ethylene during the culture period but their excess production inhibits cell division [100]. The adverse effect causes the mortality of cultures, and over-accumulation of ethylene induces senescence, abscission of leaves, and eventual leaf drop [101]. It is well known that Ag^+^ ions inhibit the physiological actions and production of ethylene because of its properties, easy uptake, and mobility in cells [102].

The number of reports suggested the application of AgNPs for improved regeneration; e.g., Bello-Bello et al. [91] documented positive influence of AgNPs on shoot formation in *Saccharum* spp. cv. Mex 69-290. They have reported that the better shoot induction might be due to an increase in nutrient elements suchas N, Mg, and Fe which are essential for plant growth. Similarly, a temporary immersion system with fortification of AgNPs has evoked a profuse multiplication in *V. planifolia* [33]. Mustafa et al. [64] used nano-priming of *P. vulgaris* seeds using CuNPs and AgNPs, and utilized seedling-derived hypocotyls as an explant for callus formation. They have documented that the callus formation was better when seeds were treated with AgNPs in comparison to control and CuNP treatment. In another study on *Musa* spp., it was confirmed that the addition of AgNPs into media increased the number of shoots, their length, leaf number, shoot FW/DW, and total chlorophyll content [85]. Also, AgNPs have been used at different stages such as callus formation, shoot induction, shoot multiplication, and rooting in *Musa* spp. [103] and *O. europaea* cv. Picual [104]. Three NPs, i.e., AgNPs, selenium (SeNPs), and chitosan (CSNPs), were utilized in different cultivars of *O. europaea* (Manzanillo, Picual, and Koroneiki), and AgNPs showed positive effect on shoot growth, whereas CSNPs and control media had less effectiveness and SeNPs exerted a negative effect [36]. The toxic effect of Se metal may be due to the replacement of sulfur atoms in sulfur-containing amino acids by Se, which results in changes in protein structure and function; simultaneously, it can cause oxidative stress, cellular damage and disrupt the plant’s metabolism [105,106]. The addition of nano-iron instead of traditional iron along with silver nitrate nanoparticles (AgNO_3_NPs) increased the shoot regeneration, leaf number/shoot, shoot FW and DW in *F. ananassa* cv. Ruby Gem [35]. El-Kosary et al. [34] had observed that callus formation was optimum at a higher concentration of AgNPs (500 μg/L) but a lower concentration (125 μg/L) was favorable for globular embryo formation as well as for the multiplication of embryos in *P. dactylifera* cv. Medjool and Sewi. The positive effect of AgNPs on somatic embryogenesis has been well documented in the same plant [90]. Whereas a negative effect of AgNPs was recorded in *Campomanesia rufa* (O. Berg) Nied, as a decreased shoot number was found as compared to control, no change in shoot biomass was noted [75]. The efficacy of silver thiosulfate, silver nitrate, and AgNPs was analyzed on the micropropagation of *Citrus australasica* F. Muell. and it was reported that AgNPs were less effective as compared to silver thiosulfate in terms of leaf abscission as well as shoot number and length, but were better than AgNO_3_ [101] (Table 3).

In vitro cultures of rice cells, when treated with MWCNTs, exhibited decreased cell density at higher concentration of NPs, and it was suggested that this might be due to self-defense response [107]. Later on, Taha et al. [89] reported a positive effect of MWCNTs on somatic embryo germination and elongation in *P. dactylifera*. In accordance, the positive influence of MWCNTs on the nodal culture of *Simmondsia chinensis* (Link) Schneider has been well documented [92]. In in vitro cultures of *O. sativa* ssp. *indica* cv. KDML105, a comparison of activated charcoal and nanocarbon on callusing and plant regeneration was carried out, which confirmed that the addition of nanocarbon proved better for callus induction frequency, its size, callus FW/DW, and ratio of no. of seedlings to calli [108].

**Table 3:** Effect of various NPs on in vitro morphogenesis in crops.

Plant	Explant	Nanoparticle (NP) Treatment	Callus/Number of Shoots or SEs/Explant (% Response)	Shoot Length (cm)	Root Induction Media	Number of Roots/Explant (% Response)	Root Length (cm)	Effect	Reference
*Alternanthera sessilis*	Node	GFAgNPs (2.0 mg/L)	153.6 ± 2.3 (100%)	-	-	-	-	In vitro cultures are genetically uniform with mother plant	[109]
*Brassica napus* cv. Hayola 401	Hypocotyl	ZnONPs (10 mg/L)	Callus- 300 mg (FW), 29 mg (DW) (88%)	-	-	-	-	Improved callus FW and DW	[110]
*Brassica nigra*	Stem	ZnONPs (1 mg/L)	Callus- 11.95 ± 1.7 g (FW) 0.70 ± 0.2 g (DW)	-	-	-	-	Induced roots and few shoots from callus, decreased FW and DW of callus	[65]
*Brassica nigra*	Leaf and stem	CuONPs (1 mg/L)	Callus (leaf)- 8.5 ± 1.9 g (FW), 0.3 ± 0.01 g (DW); Callus (stem)- 8.7 ± 1.8 g (FW), 0.4 ± 0.06 g (DW)	-	-	-	-	Decreased FW and DW of callus	[5]
*Campomanesia rufa*	Node	AgNPs (1.54 mg/L)	17	1.1	-	-	-	Less shoots in presence of NPs, but no significance difference in fresh mass of the shoots	[75]
*Cicer arietinum*	Embryo axes (EA) and embryo axes with adjacent part of cotyledon (EXC)	IONPs (15 mg/L)	EA- 51.6 ± 0.9 (86%), EXC- 53.0 ± 1.5 (88%) (var. Punjab-Noor 09); EA- 47.4 ± 0.4 (79%), EXC- 45.7 ± 2.5 (76%) (var. Bittle-98)	EA- 9.9 ± 0.3, EXC- 11.8 ± 0.5 (var. Pujab-Noor 09); EA- 7.5 ± 0.3, EXC- 8.5 ± 0.4 (var. Bittle-98)	IONPs (15 mg/L)	EA- 45.0 ± 1.2 (75%), EXC- 49.8 ± 0.9 (83%) (var. Pujab-Noor 09); EA- 41.5 ± 1.5 (69%), EXC- 47.0 ± 2.6 (78%) (var. Bittle-98)	-	Higher iron content	[111]
*Citrus australasica*	Node	AgNPs (40 μM)	17.4	3.53 ± 0.02	-	-	-	Less shoot regeneration	[101]
*Daucus carota* cv. Berlicum	Hypocotyl	Fe_3_O_4_NPs (4.02 mg/L)	-	-	-	-	-	Decreased SEs formation, mitotic index of cell culture	[112]
*Fragaria* × *ananassa* cv. Queen Elisa	Runner tips	FeNPs (0.8 ppm)	4 (Branch number)	2.80 ± 0.03 (Branch length)	-	-	3.40 ± 0.20	Increased biomass, higher percentage of relative water content (RWC), and membrane stability index (MSI)	[81]
*Fragaria* × *ananassa* cv. Ruby Gem	Runner tips	AgNO_3_NPs (10 mg/L)	11.00	4.17	-	-	-	Increasedpercentage of open buds, shoot regeneration, leaf number, shoots FW and DW	[35]
*Fragaria* × *ananassa*	Leaf	Shoot induction- Explants were treated with 200 mg/L AgNPs solution (20 min), shoot multiplication- AgNPs (0.20 mg/L)	Regeneration- 21.00 (64.44%), multiplication- 12.67 (100%)	3.93	AgNPs (0.50 mg/L)	6.67	3.40	Increased regeneration and rooting response, and biomass of plants	[113]
*Hordeum vulgare* cv. Nosrat	Mature embryos	TiO_2_NPs (60 µg/mL)	-	Callus diameter- 21 mm^2^	-	-	-	Increased number and size of callus	[42]
*Linum usitatissimum* ‘Blue di Riga’	Stem of in vitro seedling	CNPs (1×10^−3^ g/L)	Callus- 83%, 0.5 ± 0.1 g (FW), indirect embryogenesis- 25%	Callus diameter- 8.5 ± 0.3 mm	-	-	-	Reduce callus formation, embryogenesis, and organogenesis	[114]
*Linum usitatissimum*	Stem of in vitro seedling	BAP (1 mg/L) (coated with AgNPs or AuNPs)	Callus regeneration zone- 1.40 ± 0.65, rhizogenesis (50%), embryogenesis (50%) (AgNPs); Callus regeneration zone- 3.40 ± 1.22, rhizogenesis (30%), embryogenesis (70%) (AuNPs)	Callus length and width- 5.38 ± 1.30 and 5.00 ± 2.14 mm (AgNPs), 8.38 ± 1.60 and 5.38 ± 1.06 (AuNPs)	-	-	-	Increased callus formation and embryogenesis	[99]
*Linum usitatissimum*	Stem of in vitro seedling	Experiment A- Fe_3_O_4_NPs (1.5 mg/L), Experiment B- Fe_3_O_4_NPs (1 mg/L)	Experiment A- 100% somatic embryogenesis, Experiment B- 100% rhizogenesis	Callus length and width- 1.4 ± 0.38 and 1.11 ± 0.26 cm (Exp. A), 1.17 ± 0.55 and 0.98 ± 0.32 cm (Exp. B)	-	-	-	Increased callus size and embryogenesis, NPs induced genotoxicity incallus cultures	[115]
*Maerua oblongifolia*	Node	AgNPs (20 mg/L)	16.67 ± 0.57	10.43 ± 0.45	-	-	-	Increased shoot number and length, leaf number, shoot FW and DW	[83]
*Mentha longifolia*	Node	CuNPs (0.5 mg/L)	-	6.83 ± 0.74	-	-	-	Increased regeneration and shoot formation	[116]
*Musa paradisiacal* cv. Grand Nain	Shoot tip (Suckers)	ZnNPs/ZnONPs (100 mg/L)	Callus- 92%, shoot- 2.5 (92%)	-	ZnNPs/ZnONPs (100 mg/L)	6.57 (89%)	2.93	Reduced contamination and increased callus formation, shoot regeneration, shoots and roots FW, and rooting	[43]
*Musa paradisiacal*	Shoot tip (Suckers)	FCNTs (100 µg/mL)	12.5	5.2	-	-	-	Increased shoot formation	[117]
*Musa* spp.	In vitro shoot tip	AgNPs (1 ppm)	8.40	2.45	AgNPs (3 ppm)	7.10	7.70	Increased number of shoot, its length, leaf number, shoot FW and DW	[85]
*Musa* spp.	Pseudo-stem	Callus formation- AgNPs (8 ppm), shoot regeneration- AgNPs (4 ppm), multiplication- AgNPs (6 ppm)	Callus- 97.78%, multiplication coefficient- 4.22 (100%)	3.44	AgNPs (4 ppm)	5.22 (98.33%)	4.26	Increased callus formation, shoot induction and multiplication as well as rooting response	[103]
*Olea europaea* cv. Picual	Node	AgNPs (5 mg/L)	1.72	5.44	-	-	-	Increased bud sprouting, shoot length, shoot number, and number of leaves/shoot	[104]
*Olea europaea*	Node	AgNPs (10 mg/L)	4.3 ± 0.17 (cv. Manzanillo); 4.0 ± 0.00 (cv. Picual); 5.0 ± 0.00 (cv. Koroneiki)	7.0 ± 0.00 (cv. Manzanillo); 8.0 ± 0.57 (cv. Picual); 10.0 ± 0.00 (cv. Koroneiki)	-	-	-	Higher number of shoots, shoot length, leaf number, and multiplication rate	[36]
*Oryza sativa* cv. KDML105	Seed	TiO_2_NPs (25 mg/L)	2.80 ± 0.03 (56.46 ± 0.82%)	-	-	-	-	Better regeneration	[118]
*Oryza sativa*	Seed	Callus- CuONPs (10 mg/L), regeneration- CuONPs (20 mg/L) (var. Basmati 2000, Basmati 370, Basmati 385); Callus- CuONPs (10 mg/L), regeneration- CuONPs (15 mg/L) (var. Super Basmati)	Callus- 74%, regeneration- 80% (var. Basmati 2000); Callus- 86%, regeneration- 42% (var. Basmati 370); Callus- 90%, regeneration- 92% (var. Basmati 385); Callus- 94%, regeneration- 65% (var. Super Basmati)	-	-	-	-	Increased callogenesis and regeneration	[119]
*Oryza sativa* ssp. *indica* cv. RD49	Seed	TiO_2_NPs (20 mg/L)	Callus- 97.73 ± 0.17%, regeneration- 67%	-	-	-	-	Better regeneration	[120]
*Oryza sativa* ssp. *indica*	Seed	Callus- TiO_2_NPs (50 mg/L), regeneration- TiO_2_NPs (40 mg/L)	Callus- 94.67 ± 1.01%, regeneration- 3.11 (61.89 ± 1.13%) (cv. Suphanburi1); Callus- 93.25 ± 1.02%, regeneration- 3.06 (60.25 ± 1.13%) (cv. Suphanburi90)	-	-	-	-	Better regeneration	[121]
*Oryza sativa* cv. KDML105	Seed	Callus- NCNPs (5 mg/L), regeneration- NCNPs (20 mg/L)	Callus- 94.70 ± 0.86%, regeneration- 3.16 ± 0.04 (62.75 ± 0.89%)	-	-	-	-	Increased callus frequency, FW and DW, ratio of no. of seedlings to no. of regenerated calli	[108]
*Oryza sativa* cv. IR64	Seeds	Callus- AgNPs (10 mg/L), regeneration- AgNPs (5 mg/L)	Callus- 82.4 ± 5.2%, regeneration- 61 ± 6.3%	-	AgNPs (10 mg/L)	11.2 ± 0.6	4.9 ± 0.3	Increased regeneration and rooting	[88]
*Panicum virgatum*	Seed/ internode	ZnONPs (20 and 30 mg/L)	Callus induction- 90% (seed), 96% (internode), shoot regeneration- 23.10 ± 2.1 (76%) (seed), 24.00 ± 0.01 (80%) (internode)	-	-	-	-	Enhanced plant growth and development	[122]
*Phaseolus vulgaris*	Hypocotyl	AgNPs (50 mg/mL)	Callus- 97%	-	-	-	-	Increased callus formation, FW and DW	[64]
*Phoenix dactylifera*	Leaflets	Callus—MWCNTs (0.05 mg/L), SE formation and elongation—MWCNTs (0.1 mg/L)	Callus- 3.80 g, SE- 24.0	4.3	MWCNTs (0.1 mg/L)	5.3	6.0	Increased embryogenesis and elongation of shoots	[89]
*Phoenix dactylifera*	Immature inflorescences	Callus establishment- AgNPs (500 μg/L), callus differentiation- AgNPs (125 μg/L), SE formation- AgNPs (125 μg/L) (cv. Medjool); Callus establishment- AgNPs (500 μg/L), callus differentiation- AgNPs (500 μg/L), SE formation- AgNPs (125 μg/L)(cv. Sewi)	Callus- 76.66%, globular SE- 16.00, direct SE- 68.33%, germination- 0.81, multiplication- 1.00 (cv. Medjool); Callus- 73.33% globular SE- 17.33, direct SE- 68.33%, germination- 0.92, multiplication- 1.00 (cv. Sewi)	-	-	-	-	Increased SE formation	[34]
*Phoenix dactylifera* cv. Hayani	Shoot tip	AgNPs (1 mL/L)	Callus- 4.60 g (FW), SE initiation- 9.39, SE development- 35.30	1.80	-	-	-	Increased SE length, no. and length of leaves, shoots were genetically uniform	[90]
*Rhizoma polygonati*	Tuber	Fe_3_O_4_NPs (0.4 mg/L)	4	-	Fe_3_O_4_NPs (0.5 mg/L)	9.5	-	Increased number of shoots and roots	[123]
*Rubus adenotrichos*	In vitro shoot	-	-	-	SWCNTs-COOH (4 µg/mL)	8.60 ± 5.75	0.6	Increased rooting of shoots and growth of plants	[124]
*Saccharum* spp.cv. Mex 69-290	In vitro Shoot	AgNPs (50 mg/L)	47.28 ± 1.69	5.55 ± 0.24	-	-	-	Improved regeneration and shoot length in temporary immersion bioreactors	[91]
*Simmondsia chinensis*	Node	MWCNTs (0.002 g/L)	16.00	1.36	-	-	-	Improved regeneration	[92]
*Solanum lycopersicon*	Hypocotyl (For callus), cotyledonary nodes (For regeneration)	Fe_3_O_4_NPs (3 mg/L)	Callus- 64.26 ± 0.38%, shoot- 8.2 ± 0.09 (cv. Nora); Callus- 83.28 ± 0.94%, shoot- 10.8 ± 0.09 (cv. PS-10); Callus- 74.48 ± 0.39%, shoot- 9.7 ± 0.09 (cv. Peto); Callus- 56.32 ± 0.47%, shoot- 6.6 ± 0.12 (cv. Roma)	-	-	-	-	Better callus and shoot formationshowing resistance to salinity stress	[93]
*Solanum lycopersicum* cv. Edkawy	Cotyledon	ZnONPs (15 mg/L)	Regeneration- 83.34 ± 0.23% (cv. Edkawy), 64.58 ± 0.15% (cv. Anna Aasa), 78.16 ± 0.23% (cv. Australische Rosen), 67.7 ± 0.47% (cv. Sankt Ignatius), 87.64 ± 0.58% (cv. Sandpoint)	-	-	-	-	Improved salinity stress and regeneration frequency	[4]
*Solanum tuberosum*	Leaf	SiO_2_NPs (50 mg/L)	Callus- 1.1 g (FW), 0.07 g (DW) (cv. Proventa); Callus- 1 g (FW), 0.05 g (DW) (cv. Sante)	9 (cv. Proventa); 8 (cv. Sante)	-	6 (cv. Proventa); 4 (cv. Sante)	10 (cv. Proventa); 8 (cv. Sante)	Increased resistance to salinity stress in terms of various morphological traits	[94]
*Solanum tuberosum* cv. White Desiree	Node	AgNPs (2 mg/L)	-	7.8	-	-	12	Increased shoot and root DW, root length and leaf area, decreased shoot length	[95]
*Solanum tuberosum* cv. Spunta	Sprout	CSNPs (250 mg/L)	90.97 ± 1.41	12.40 ± 0.38	-	-	-	Production of potato virus Y (PVY) free plants	[125]
*Triticum aestivum*	Mature embryo	Callus- 1X-3X of all NPs (ZnO/CuO/γ-Fe_3_O_4_), embryogenic callus- 1x (CuO/γ-Fe_3_O_4_), SE formation- 3X γ-Fe_3_O_4_NPs (genotypeKırik); Callus- 2XZnONPs/3XZnONPs/3XCuONPs, embryogenic callus-3XZnONPs, regeneration- 3X ZnONPs (genotype ES-26)	Callus- 100%, embryogenic callus- 97.5%, SEs- 1.69, plantlet- 9.00 (genotypeKırik); Callus- 97.50%, embryogenic callus- 41.38%, SE- 1.70, plantlet- 6.75 (genotype ES-26)	-	-	-	-	Genotype Kırik: higher callus, SE and plantlet formation; Genotype ES-26: same frequency of callus but less SE and plantlet formation	[126]
*Triticum aestivum*	Mature embryo	CuNPs (0.015 mg/L) + AgNPs (4 mg/L)	Callus- 90.00%, embryogenic callus- 84.67%, regeneration- 71.67% (genotype AS-2002); Callus- 95.00%, embryogenic callus- 78.00%, regeneration- 68.33% (genotype Wafaq-2001)	-	-	-	-	Increased callus and regeneration frequency	[127]
*Vanilla planifolia*	In vitro shoot	AgNPs (50 mg/L)	14.89 ± 0.40	4.71 ± 0.23	-	-	-	Increased regeneration, shoot length, and biomass	[33]
*Vigna unguiculata* cv. Ülkem	Plumule of embryo	MgONPs (555 mg/L)	10.00 (82.50%)	1.45	MgONPs (370 mg/L)	0.75 (22.50%)	0.72	Increased shoot number, frequency, and rooting response	[128]

AgNPs: silver nanoparticles; AgNO_3_: silver nitrate; AgNO_3_NPs: silver nitrate nanoparticles; AuNPs: gold nanoparticles; CH-ZnO: chitosan–zinc oxide nano-bioformulation; CNPs: carbon nanoparticles; CSNPs: chitosan nanoparticles; CuNPs: copper nanoparticles; CuONPs: copper oxide nanoparticles; DW: dry weight; FCNTs: functionalized carbon nanotubes; FeNPs: iron nanoparticles; Fe_3_O_4_NPs: iron oxide nanoparticles; FW: fresh weight; GFAgNPs: *Gracilariafoliifera* coated silver nanoparticles; IONPs: iron oxide nanoparticles; MgONPs: magnesium oxide nanoparticles; MWCNTs: multi-walled carbon nanotubes; NCNPs: nanocarbon nanoparticles; SE(s): somatic embryo(s); SiO_2_NPs: silicon dioxide nanoparticles; SWCNTs: single-walled carbon nanotubes; TiO_2_NPs: titanium dioxide nanoparticles; ZnNPs: zinc nanoparticles; ZnONPs: zinc oxide nanoparticles.

### 4.2. Metal Oxide NPs

Metal oxide NPs are another type of NP that has proven results on many crops (Table 3). In *O. sativa* ssp. *indica* cv. KDML105, comparisons of two NPs (ZnO and TiO_2_) suggested that ZnONPs showed toxic effects whereas TiO_2_NPs enhanced the regeneration frequency [118]. Later on, Chutipaijit and Sutjaritvorakul [121] also documented a positive influence of TiO_2_NPs on the indirect regeneration of rice cultivars Suphanburi1 and Suphanburi90. Zafar et al. [65] observed the negative influence of ZnONPs on *B. nigra* stem explant as only a few shoots and roots were emerged from the callus. Later on, when the CuONP fortification of NPs in media was performed, it caused root emergence from callus from both leaf and stem explants of *B. nigra*; also, the biochemical potency of the roots which were emerged from the callus was different as compared to the seedlings, and this is due to lower concentrations of NPs used for leaf and stem explants as compared to seeds [5]. Similar to rice cultivar KDML105, TiO_2_NPs displayed a beneficial effect as compared to ZnONPs on callus induction and plant regeneration in another cultivar RD49 [120]. Later on, the negative effect of ZnONPs on *N. tabacum* cv. Bright Yellow-2 (BY-2) cells with respect to viability, packed cell volume, and FWs were also reported. There was a significant decrease in mitotic index and changes in cell structure such as endoplasmic reticulum, mitochondrial dysfunction, and Golgi apparatus, along with an increase in ROS and reactive nitrogen species (RNS) [87]. Further, to evaluate the programmed cell death, an increase in plasma membrane integrity, and activities of protease, caspase-like, and acid phosphatases were observed along with nuclear cell morphology and DNA fragmentation, suggesting the phytotoxic effect of ZnONPs. Some studies suggested that metal and metal oxide NPs showed similar effects on cultures, e.g., zinc nanoparticles (ZnNPs) and ZnONPs both increased regeneration and rooting responses in *Musa paradisiacal* L. Upon analysis, it was confirmed that treatment with both types of NPs elevated total proline and chlorophyll contents as well as increased the activities of antioxidant enzymes in shoots [43]. The comparison between ZnO bulk and NPs on *B. napus* cv. Hayola 401 showed that the application of ZnONPs improved the formation of calli in terms of FW and DW in comparison to bulk ZnO [110]. Irum et al. [111] documented that the callus of *C. arietinum* var. Punjab-Noor 09 and Bittle-98 showed good callus proliferation from embryo axes and embryo axes with the adjacent parts of cotyledon explant on media containingiron oxide nanoparticles (IONPs) in comparison to control. However, transferring this callus on regeneration medium showed only an increase in callus size which failed to undergo redifferentiation.

The effect of iron oxide nanoparticles (Fe_3_O_4_NPs) on hypocotyls of *Daucus carota* L. cv. Berlicum revealed that its lower concentrations facilitated somatic embryo formation, but higher concentrations ceased its differentiation [112]. Li et al. [123] compared Fe_3_O_4_NPs and micro-cube on the in vitro morphogenic response of *Rhizoma polygonati* Odorati, and confirmed the positive influence of the former than later on shoot and root formation. In *L. usitatissimum* cultures, augmentation of the medium with Fe_3_O_4_NPs during the culture initiation phase induced somatic embryogenesis, but its addition after callus formation led to rhizogenesis [115]. An interesting study was carried out on two genotypes of *Triticum aestivum* L. Kırik and ES-26, where Fe, Cu, and Zn of MS media were replaced by NP versions of elements (ZnO, CuO, and γ-Fe_3_O_4_) in the same concentration (1X) and two and three times higher amount (2X and 3X). The results suggested that the overall response in terms of callus, somatic embryo, and plantlet formation was less for the ES-26 genotype in comparison to the control and Kırik genotypes, suggesting variation in NPs’ effect between the genotypes [126]. In the same way, Malik et al. [127] compared CuSO_4_ and AgNO_3_ with their NP counterpart and evaluated their effect on the in vitro response of two genotypes of *T. aestivum* cv. AS-2002 and Wafaq-2001. They have suggested that the combined application of both the NPs was more beneficial than their individual usage, also the response was better for cv. AS-2002 than Wafaq-2001. The majority of studies suggested that the enhancement in growths of plants in response to NPs might be due to the up-regulation or down-regulation of different hormonal pathways, especially the cytokinin, which evoked culture growth [110].

### 4.3. Role of Green NPs

Rapid progress in the field of nanotechnology has enabled the synthesis of NPs of different types, sizes, and morphologies; and, NPs generated using plants are reported to have a less toxic and more stable effects [129]. CuONPs synthesized utilizing *Azadirachta indica* leaf extracts were used to evaluate its effect on four varieties of *O. sativa* (Super Basmati, Basmati 2000, Basmati 370, and Basmati 385). They have suggested that the augmentation of NPs in the media improved callogenesis and organogenesis; however, variation has been detected between the varieties [119]. In another study, AgNPs coated with marine red alga *Gracilaria foliifera* (GFAgNPs) showed growth-stimulating properties in comparison to the traditionally used hormones like 6-benzylaminopurine (BAP) and kinetin (Kn) on *Alternanthera sessilis* L. regeneration [109]. In line with this, AgNPs synthesized using *Parthenium hysterophorus* extract when augmented into the media have promoted callus formation, shoot regeneration, and rhizogenesis in *O. sativa* cv. IR64, and also suggested the inhibitory effect of AgNPs on ethylene perception [88]. Likewise, AgNPs synthesized using leaf extract of *Ochradenus arabicus* increased the shoot number, length, FW, DW, chlorophyll, total protein, and total proline contents as well as activities of enzymes like SOD and CAT in cultures of *Maerua oblongifolia* (Forssk.) A. Rich [83]. Green synthesis of ZnONPs using *Cymbopogon citrates* extract enhanced callus induction and regeneration from seed and internode explants of *Panicum virgatum* L. [122]. They have observed that ZnONPs have a positive influence on both the explant, but a 20 mg/L concentration was suitable for the seed explant whereas, for the internode explant, 30 mg/L was better. In addition, the application of manganese oxide nanoparticles (MgONPs) synthesized using walnut shell extract increased the regeneration response in *Vigna unguiculata* L. Walp cv. Ülkem [128] (Table 3).

## 5. Ramifications of NPs on In Vitro Cultures

### 5.1. NPs to Mitigate Stress and Virus Resistance

The application of nanomaterials can help in improving tolerance against biotic and abiotic stresses via in vitro cultures that help in the micropropagation of stress-resistant crops. In five cultivars of *S. lycopersicum*, the salinity stress provided using NaCl was used to evaluate the effect of ZnONPs, and results showed that the treatment significantly improved callus growth and regeneration frequency (Table 3), as well as the content of different elements, protein, and antioxidant enzymes (Table 2), which justifies the resistance towards salinity-induced stress [4]. In another study to improve salinity stress in potato cv. Sante and Proventa, Gowayed et al. [94] utilized silicon dioxide nanoparticles (SiO_2_NPs) and observed better morphological traits when SiO_2_NPs along with NaCl were used, signifying the improved plant growth under salinity stress. A comparative study was carried out in four cultivars of *S. lycopersicon* in which the effect of NPs like Fe_3_O_4_ and ZnO was seen and it was observed that the aforesaid treatments significantly eliminated the salinity stress in callus and shoot formation, and the maximum response for cv. PS-10 and least for cv. Roma was recorded [93]. Drought stress has been a serious concern for the crops and thus in *F. ananassa*, a combination of iron nanoparticles (FeNPs, 0.8 ppm) + salicylic acid (SA, 0.01 mM) proved beneficial as an increased biomass of plantlets with a higher percentage of relative water content (RWC) and membrane stability index (MSI) was observed, which confirmed that the cultures withstand drought stress [81]. Another concern for crop productivity is infection with virus, and potato virus Y (PVY) causes serious loss in the yield and quality of potatoes. Recently, to overcome this, Elsahhar et al. [125] evaluated the role of CSNPs and suggested that their treatment proved effective in producing virus-free plants.

### 5.2. NPs’ Influence on Induction of Somaclonal Variation

The addition of NPs in the culture media is known to affect the plant cells at biochemical and/or molecular levels; thus, there are chances that it might produce somaclonal variations. Somaclonal variation is one of the consequences of in vitro cultures which is associated with any changes in chromosome number, chromosome structure, DNA sequence, DNA methylation, mitotic crossing over, and activation of transposable elements [130]. However, it has advantages as well as disadvantages, and the main advantages are the development of useful characteristics like plant size, flower colour, leaf variegation, fruit ripening, resistance to biotic and abiotic stresses, and elevated secondary metabolites production [131]. Sometimes, the higher concentrations of NPs cause toxicity in plants which affects the mitotic index, DNA integrity, and alters the protein and DNA expression [54]. Some studies reported the change in ploidy levels, e.g., *L. usitatissimum* calli grown on media containing carbon NPs (CNPs) showed an increased number of tetraploid cells and level of DNA methylation [114]. In another study, Kokina et al. [132] observed a high rate of somaclonal variation in calli and regenerated shoots of *L. usitatissimum* when the medium consisted of AuNPs and less variation in the presence of AgNPs. Hence, it has been suggested that the regenerated plants need to be investigated if the study aims to induce true-to-type plants.

### 5.3. NPs as an Elicitor for In Vitro Production of Secondary Metabolites

Earlier studies have suggested that NPs act as signal components and modify the physiological and metabolic responses of plants. This has opened an alternative strategy for the production of targeted secondary metabolites in plant cell cultures using NPs as an elicitor [133]. The effectiveness of NPs is due to their small size, as they can easily attach to the plant cell walls, destroy them or change their permeability, and thus significantly affect the cellular metabolism [134]. This is due to the dual role played by NPs, first by acting as an efficient nutrient and second by acting as an elicitor, which enhances secondary metabolite production [135]. Recently, the exploitation of NPs for the production of economically and commercially important secondary metabolites from crop species has been well documented (Table 4). Al-Oubaidi and Mohammed-Ameen [136] found that AgNO_3_NPs increased callus formation in *Calendula officinalis* L. at a 0.3 mg/L concentration, but its higher concentration (1.2 mg/L) favored the synthesis of various essential oils. Hairy root cultures are one of the promising ways for secondary metabolite production and in *Datura metel* L. hairy roots, the addition of AgNPs proved beneficial for the enhancement of biomass as well as atropine content in comparison to AgNO_3_ and biotic elicitors (*Bacillus cereus* and *Staphylococcus aureus*) [137]. In *Cucumis anguria* L. hairy roots, AgNPs significantly elevated the content of different metabolites in comparison to its bulk counterpart (AgNO_3_) [80]. In a cell suspension of *Corylus avellana* L. cv. Gerd Eshkevar, augmentation of media with AgNPs increased the taxol [78] as well as taxane contents [79]. AgNPs obtained via green synthesis using an extract of *Bacillus marisflavi* increased the contents of different groups of compounds like hydroxybenzoic acids, hydroxycinnamic acids, and flavonols in the cell suspension of *Momordica charantia* L. [84]. A comparison between AuNPs and AgNPs has been carried out for the shoot culture of *Lavandula angustifolia* Mill. cv. Munstead where shoots grown in the presence of both NPs significantly affected the composition of essential oil. Their addition in media decreased the content of low-molecular-weight compounds (α- and β-pinene, camphene, δ-3-carene, *p*-cymene, 1,8-cineole, trans-pinocarveol, and camphoriborneol), which were replaced with high-molecular-weight compounds (τ- and α-cadinol 9-cedranone, cadalene, α-bisabolol, cis-14-nor-muurol-5-en-4-one, and (*E*,*E*)-farnesol) [138]. In callus cultures of *Allium sativum* L., the contents of allicin, di-allyldisulfide, and vinyldithiin have been elicited using AgNPs and NaCl [139].

The addition of CuNPs and cobalt nanoparticles (CoNPs) in shoot cultures of *Mentha longifolia* L. revealed that they have positively influenced linalool synthesis and negatively affected linalyl acetate content. Their results also confirmed that CuNPs were better for regeneration, but maximum essential oil synthesis was observed in the presence of CoNPs [116]. Contrarily, CuONPs significantly enhanced the contents of glucosinolates, phenolic compounds, hydroxy-benzoic acids, hydroxycinnamic acids, and flavonols in hairy roots of *B. rapa* spp. *pekinensis*. They have also confirmed the up-regulation of different pathway genes such as *MYB34*, *MYB122*, *MYB28*, *MYB29*, *PAL*, *CHI*, and *FLS* after the exposure of hairy roots with NPs [140]. Al-Oubaidi and Al-Khafagi [141] compared the effectiveness of MgONPs and CuONPs on *Punica granatum* L. callus cultures for metabolite synthesis, and reported that the contents of metabolites varied according to concentration and type of NPs augmented in the media. They have observed that the level of gallic acid, tannic acid, ellagic acid, chlorogenic acid, acacetin, cinnamic acid, and geniste in was increased in the presence of MgONPs, whereas CuONPs elevated the levels of brevifolincarboxylic acid, catechin, rutin, coumaric acid, ferulic acid, benzoic acid, and kaempferol. Al-Khafagi and Al-Oubaidi [142] studied the contents of similar metabolites of the same species after treatment using NPs on shoot tip culture. However, their results differed as compared to an earlier report as the majority of the compounds were elicited after MgONP treatment except for catechin and kaempferol which were increased with CuONPs. Similarly, TiO_2_NPs stimulated higher lignin content in the cell suspension of *L. usitatissimum* cv. Kerman Shahdad as compared to ZnONPs [82]. In another cultivar (Barbara) of *L. usitatissimum*, different lignans (secoisolariciresinoldiglucoside and lariciresinoldiglucoside) and neolignans (dehydrodiconiferyl alcohol glucoside and guaiacylglycerol-β-coniferyl alcohol ether glucoside) were detected in seedlings and calli, and it was noted that a higher concentration (500 mg/L) of ZnONPs proved beneficial in elevating the contents in seedlings, whereas a lower concentration (10 mg/L) favoured metabolite synthesis in callus [68]. The effectiveness of the chitosan–zinc oxide nano-bioformulation (CH-ZnO) combination proved better in comparison to ZnONPs not only for callus biomass but also for tannin content, whereas nicotine content was better when ZnONPs were used in media containing leaf-derived callus of *Nicotiana benthamiana* Domin [86]. In a recent study, CuO and ZnO NPs were synthesized using *Nigella sativa* L. extract, and their effect on three varieties of *V. radiate* (var. NCM-13, MgAT-7, and MgAT-4) was assessed. Their results confirmed that both the NPs affected different metabolites in all three varieties as increased phenolics were recorded in the presence of CuONPs, whereas increased glycoside was detected in the presence of ZnONPs [135]. Recently, Abu-Al hayl and Al-Oubaidi [143] carried out an experiment using SiO_2_NPs on the callus of *Tagetes erecta* L. where amounts of gallic acid, syringic acid, ellagic acid, quercetin, quercetagetin, lutein, and kaempferol increased significantly at a higher concentration in comparison to the lower concentration of NPs (Table 4).

**Table 4:** Effects of various NPs on elicitation of metabolites in in vitro cultures of different crops.

Plant	Metabolite(s)	Culture Type	Nanoparticle (NP) Treatment and Time	Remarks	Reference
*Allium sativum*	Allicin, di-allyldisulfide and vinyldithiin	Callus	AgNPs (2 mg/L), time- 4 w	Increased content of all metabolites	[139]
*Brassica rapa* spp. *pekinensis*	Glucosinolates (gluconasturtiin, glucobrassicin, 4-methoxyglucobrassicin, neoglucobrassicin, 4-hydroxyglucobrassicin, glucoallysin, glucobrassicanapin, sinigrin, progoitrin, and gluconapin), phenolic compounds (flavonols, hydroxybenzoic and hydroxycinnamic acids), hydroxy-benzoic acids (vanillin, *p*-hydroxybenzoic, protocatechuic, syringic, gentisic acids), hydroxycinnamic acids (chlorogenic, *p*-coumaric, ferulic, and *t*-cinnamic acids) and flavonols (myricetin, quercetin, catechin, kaempferol, rutin, naringenin and hesperidin)	Hairy roots	CuONPs (100 mg/L), time- 48 h	Increased content of all metabolites and expression of genes	[140]
*Calendula officinalis*	α-Pinene, β-pinene, ρ-cymene, α-thujene, calendulaglycoside, α-cadinene, cadinol, *t*-muurolol, 1,8-cineole and limonene	Callus	AgNO_3_NPs (1.2 mg/L), time- 4 w	Increased contents	[136]
*Capsicum annum* and *C. frutescens*	Capsaicin	Cell suspension	AgNO_3_NPs (3 mg/L), time- 6 d	Increased content	[144]
*Corylus avellana* cv. Gerd Eshkevar	Taxol	Cell suspension	AgNPs (5 ppm), time- 1 w	Increased content, decreased cell viability	[78]
*Corylus avellana* cv. Gerd Eshkevar	Taxol and baccatin III	Cell suspension	AgNPs (5 ppm), time- 24 h	Increased contents	[79]
*Cucumis anguria*	Hydroxybenzoic acids (*p*-Hydroxybenzoic acid, gallic acid, protocatechuic acid, syringic acid, gentisic acid, salicylic acid, vanillic acid, β-resorcylic acid, hydroxycinnamic acids (Caffeic acid, *p*-coumaric acid, *o*-coumaric acid, ferulic acid, chlorogenic acid, *t-*cinnamic acid), flavonols (Myricetin, quercetin, kaempferol, catechin, rutin, naringenin, biochanin A), phenolics (vanillin, veratric acid, homogentisic acid, hesperidin)	Hairy root	AgNPs (1 mg/L), time- 21 d	Increased biomass and content	[80]
*Datura metel*	Atropine	Hairy roots	Nanosilver (conc.- NM), time- 48 h	Increased biomass and content	[137]
*Lavandula* *angustifolia* cv. Munstead	Essential oils	Shoots	AgNPs and AuNPs (10 mg/dm^3^), time- NM	Decreased content of low-molecular-weight compounds (e.g., α- and β-pinene, camphene, δ-3-carene, *p*-cymene, 1,8-cineole, trans-pinocarveol, camphoriborneol), and increased content of high-molecular-weight compounds (τ- and α-cadinol 9-cedranone, cadalene, α-bisabolol, cis-14-nor-muurol-5-en-4-one, (*E*,*E*)-farnesol)	[138]
*Linum* *usitatissimum* cv. Kerman Shahdad	Lignan	Cell suspension	TiO_2_NPs (150 mg/L), time- 72 h	Increased content	[82]
*Linum* *usitatissimum* cv. Barbara	Lignans (secoisolariciresinoldiglucoside, lariciresinoldiglucoside) and neolignans (dehydrodiconiferyl alcohol glucoside and guaiacylglycerol-β-coniferyl alcohol ether glucoside)	Seedlings and callus	ZnONPs (500 mg/L)/(10 mg/L), time- 30 d	Higher ZnONPs increased contents in seedling; lower ZnONPs increased contents in callus	[68]
*Mentha longifolia*	Essential oils (Linalool and linalyl acetate)	Shoots	CoNPs (0.8 mg/L), time- 30 d	Increased linalool and decreased linalyl acetate contents	[116]
*Momordica* *charantia*	Hydroxybenzoic acids (*p*-Hydroxybenzoic acid, gallic acid, protocatechuic acid, syringic acid, gentisic acid, salicylic acid, vanillic acid, β-resorcylic acid), hydroxycinnamic acids (Caffeic acid, *p*-coumaric acid, *o*-coumaric acid, ferulic acid, chlorogenic acid, *t-*cinnamic acid), flavonols (Myricetin, quercetin, kaempferol, catechin, rutin, naringenin, biochanin A)	Cell suspension	AgNPs (5 mg/L), time- 48 h	Increased contents	[84]
*Nicotiana* *benthamiana*	Tannin and nicotine	Callus	CH-ZnO (400 ppm)/ZnONPs (200 ppm), time- 7 d	CH-ZnOincreased tannin content; ZnONPs increased nicotine content	[86]
*Olea europaea*	Oleuropein, OH-tyrosol, ligustroside and oleacein	Shoot tips	AgNO_3_NPs (1 and 2 mg/L), time- 30 days	Increased contents	[145]
*Oryza sativa* cv. Swarna	Carotenoids	Seedling leaves	AgNPs (40 ppm), time- 14 d	Increased content	[57]
*Punica granatum*	Tannins (Gallic acid, tannic acid, ellagic acid, brevifolincarboxylic acid), phenols (chlorogenic acid, catechin, rutin, coumaric acid, ferulic acid, benzoic acid, acacetin, cinnamic acid, genistein, kaempferol)	Callus, shoot tip	MgONPs (2.5–10 mg/L)/CuONPs (5–20 mg/L), time- 21 d	Callus: MgONPs, gallic acid, tannic acid, ellagic acid, chlorogenic acid, acacetin, cinnamic acid, genistein; CuONPs: brevifolincarboxylic acid, catechin, rutin, coumaric acid, ferulic acid, benzoic acid, kaempferol; Shoot tip- MgONPs- gallic acid, tannic acid, ellagic acid, brevifolincarboxylic acid, chlorogenic acid, rutin, coumaric acid, ferulic acid, benzoic acid, acacetin, cinnamic acid, genistein; CuONPs: catechin, kaempferol	[141,142]
*Tagetes erecta*	Gallic acid, syringic acid, ellagic acid, quercetin, quercetagetin, lutein and kaempferol	Callus	SiO_2_NPs (200 mg/L), time- 30 d	Increased contents	[143]
*Vigna radiata* var. NCM-13, MgAT-7, and MgAT-4	Phenolic and glycosides	Callus and shoots	CuONPs (0.5 mg/L)/ ZnONPs (0.5 mg/L), time- NM	Overall, callus synthesized more metabolite than shoots, CuONPs-increased phenolics, ZnONPs increased glycoside content	[135]

AgNPs: silver nanoparticles; AgNO_3_NPs: silver nitrate nanoparticles; AuNPs: gold nanoparticles; CH-ZnO: chitosan–zinc oxide nano-bioformulation; CoNPs: cobalt nanoparticles; CuONPs: copper oxide nanoparticles; MgONPs: magnesium oxide nanoparticles; TiO_2_NPs: titanium dioxide nanoparticles; ZnONPs: zinc oxide nanoparticles. NM: not mentioned.

NPs, when acting as elicitors, either bind directly to elicitor-binding sites or produce endogenous messenger molecules that will bind to the sites and initiate the responses. This is due to NPs’ interaction with some of the plant cell-wall and membrane components [146]. Initially, NPs involve an active exchange of ions like Na^+^/K^+^/Cl^−^ effluxes and Ca^2+^/H^+^ influxes through the plasma membrane into the cytosol. Among all these, Ca^2+^ influxes are considered asthe most important event due to their involvement in various physiological and cellular pathways, and it plays a pivotal role in the first steps of the elicitation mechanism [147]. In another hypothesis, a cascade of events has been described where Ca^2+^ flux movements and ROS produced by oxidative burst act as messengers that led to the up-regulation/phosphorylation of mitogen-activated protein kinase (MAPK) [146] or affected ATPase activity and increase the cytoplasmic acidity, leading to metabolite synthesis [148]. The structure of the plant cell wall is consistent with the size of the NPs for entry into the cell where the ROS accumulation can be triggered [149]. This ROS will interfere with the plasma membrane and affect the permeability of the cells; thus, as a result, more NPs enter into cells, causing more stress and stimulating the production of stress-induced secondary metabolites [21]. Other studies suggested that NADPH and other oxidases also become activated through Ca^2+^ movements and they are responsible for the generation of ROS in plant cells [150,151]. This ROS generation results in the activation of cGMP-dependent protein kinase and the phosphorylation of MAPKs, which results in transcriptional reprogramming events of genes of secondary metabolite pathways [152]. Kohan-Baghkheirati and Geisler-Lee [153] stated that the G-proteins (Guanine nucleotide-binding proteins) can also activate the metabolite accumulation through de novo biosynthesis of stress-signaling compounds such as SA, jasmonic acid (JA), and methyl jasmonic acid (MeJA). Similarly, it is also suggested that the expression of genes involved in oxidative stress and the accumulation of ROS acts as a signal for metabolite synthesis [154,155].

### 5.4. NPs’ Uptake, Biochemical and Molecular Attributes in Plant Cell

The pore size of plant cell walls is usually in the range of a few nanometers which acts as a barrier to foreign materials [156]. However, the diameters of NPs are usually smaller in comparison to the diameters of the cell-wall pores; hence, they can easily penetrate and reach the plasma membrane. On the other hand, if the size of NPs is higher than the pore size, they enter the cell either by changing the size of existing pores or by inducing new larger pores in the cell wall [7]. After crossing the cell wall, NPs reach the cell membrane and are then internalized towards cytosol or other organelles either by endocytosis, specific membrane-bound transporter proteins (aquaporins), or through the induction of new pores using ion-carrier substances [157,158]. NPs help in the regulation of processes like cell signaling and the regulation of the plasma membrane; they bind with different cytoplasmic organelles and interfere with the metabolic processes at the site [159,160]. The translocation of NPs also depends on the concentration and the nature of the plant species, but usually, the passages of uptake and transportation are via the xylem [161], and it was also found that NPs followed the stomatal pathway in the leaf [162]. Further, they can be transported from one cell to another either by the apoplastic or symplastic pathway or via plasmodesmata [163,164]. Nair et al. [165] suggested that NPs, after crossing the membrane, stick with the membrane and interfere with different organelles. It has been reported in many studies that the uptake of NPs is closely associated with the absorption of moisture and nutrients from the media [166,167]. In addition, few in vitro studies have reported the uptake of NPs via clathrin-independent and -dependent pathways in *N. tabaccum* [168] or the endosomal pathway in *Catharanthus roseus* (L.) G. Don [169]. Kokina et al. [99] documented that the plant cells take up plant growth regulators (PGRs) for differentiation and redifferentiation, and the metal NPs are transported along with PGRs. It is also suggested that NP internalization becomes different in the case of cell-suspension cultures, where endocytosis in the vacuole occurs from the apoplast through vesicles formed from the plasma membrane [170,171], e.g., carbon nanotubes (CNTs) entered the cell wall through endocytosis and moved towards the cell membrane in tobacco cell suspension [172]. In comparison, NPs enter through parenchymatous intercellular spaces which assist the diffusion of liquid solution to cotyledon in seeds [173,174]. Physiologically, they affect the plant metabolism by delivering micronutrients [175], along with this, they regulate various gene functions [176] and interfere with different oxidative processes [177]. Further, they take part in electron transfer in plants, thus increasing the activity of many enzymes and influencing plant mineral nutrition [178,179]. As the size of NPs accumulates in intracellular spaces, their higher concentration renders toxicity [37,180,181]. However, it is noted that the toxic effects of NPs are dose-dependent [182], and a negative response is due to injury in the cell wall and membrane [183].

Plants induce various responses to combat stress such as the production of various ROS like singlet oxygen, superoxide, hydrogen peroxide, and hydroxyl radical, which are the main oxidative outbursts in plant cells after stress induction [177]. Depending on their concentration, ROS can work positively or negatively. At low concentration, they act as secondary messengers in intracellular signaling that induce several responses in plant cells including stress tolerance [62,184], whereas a higher concentration of ROS causes damage to biomolecules by apoptosis or necrosis, a disruption of the metabolic pathway through inactivation of the enzyme thatresults in plant cell death [185,186]. ROS are involved in many stress adaptations in plants [187,188], and therefore robust defense mechanisms have been developed by plants, viz, enzymatic (SOD, POD, CAT, APX, etc.), non-enzymatic (ascorbate, glutathione, carotenoids, tocopherols, phenolics, etc.), and antioxidant production [189]. Plants also activate the MAPK pathway that boosts the plant antioxidant elements to come in contact with ROS [190]; also, H_2_O_2_ and MDA are the measures to evaluate the stress in plants as they modulate the unstable ROS [189,191]. Proline is also a well-accepted stress marker as it has ameliorative properties suggesting its involvement in mitigating oxidative stress [192]. Its accumulation is reported to act as an antioxidant for neutralizing the toxic effects of ROS and it is also known to maintain the structure of proteins and membranes of cells [193]. Similarly, the activities of enzymes are also evaluated and the most commonly analyzed is phenylalanine ammonia-lyase (PAL). It is the first enzyme of the phenylpropanoid pathway that synthesizes many compounds which are the major protectants against stress, and hence its evaluation can be correlated with the effect of stress on plants [194]. Pigments like chlorophyll and carotenoids are attributed as precursors of abscisic acid (ABA) that modulates stress responses [195]. The loss in chlorophyll causes a surplus of electrons to combine with molecular oxygen and eventually form ROS [196]. The chlorophyll donates an electron to a series of molecular intermediates called an electron transport chain [197], whereas carotenoids are structural components of the photosynthetic antenna and reaction center complexes that protect photosynthetic organelles against harmful photo-oxidative processes [198].

The enzymes require metallic ions as a co-factor to complete the function during photosynthesis [199], and due to the physical properties of NPs, they dissociate quickly in the cytosol and aid enzymes at the cellular level to facilitate photosynthesis [200]. The addition of NPs increases the activities of enzymes like POD, CAT, and nitrate reductase, that also favours regeneration by affecting important physiological and biochemical processes [201]. Parida and Das [202] documented that the treatment of cultures with NPs induced a better chlorophyll a/b ratio indicating the activeness of PS-I and PS-II, which might be beneficial for regeneration. Various biochemical parameters are being analyzed to observe the stress imposed on plant cells or tissues after the application of various NPs (Table 2). It has been suggested that Fe and Zn stimulates the antioxidant enzyme activity in plants, and helps in the reduction of the free radical effect [203]; FeNPs are also reported to increase the gene expression of enzymes involved in photosynthesis and thus assist in enhancing the process [204]. Likewise, ZnONPs improved plant growth by affecting the electron transfer chain and increasing enzymatic antioxidants, reducing ion leakage, and improving the Hill reaction [205]. Zn also plays a vital role as a co-factor for several enzymes comprising superoxide, catalase, and dismutase, which inhibit ROS stress [206]. In seedling and callus of *L. usitatissimum* cv. Barbara, the gradual increase in ROS production was observed as the concentration of ZnONPs increased and it elevated the formation of membrane lipid peroxidation, protein carbonylation, and 8-oxo guanine [68]. Similarly, in *B. nigra* callus and seedling ZnONP treatment, increased antioxidant activity, phenolic, and flavonoid contents [65] were observed, whereas in cell suspension cultures of cultivar Kerman Shahdad of *L. usitatissimum*, increased PAL and CAD activities, and levels of total phenols [82] were observed. Likewise, in in vitro cultures of different crops like *M. paradisiacal* cv. Grand Nain [43], different cultivars of *S. lycopersicum* [4], *N. tabacum* cv. BY-2 [87], etc., augmentation of media with ZnONPs changes the biochemical parameters. In *N. benthamiana*, a combination of chitosan–zinc oxide nano-bioformulation (CH-ZnO) increased chlorophyll, carotenoid, proline contents, and enzyme (PAL and AO) activities, but decreased MDA and H_2_O_2_contents [86] (Table 2). In addition, iron oxide nanoparticles (Fe_2_O_3_NPs) changed the activities of different enzymes and antioxidant compounds in *Cichorium intybus* L. [77] and *S. lycopersicon* [93], and FeNPs in *F. ananassa* [81].

Cu is another important metal element, but at a higher level, it induces toxicity due to its binding with sulfhydryl groups in proteins which eventually inhibit enzyme activity [207]. Similarly, higher concentrations of CuONPs adversely affected the growth as Cu ions released from NPs are impermeable to the plasma membrane, thus causing a deficiency of essential nutrients [208]. CuONPs inside the cell taken up by lysosomes increase the release of Cu ions that ultimately produce intracellular ROS [209,210]. It also induces oxidative stress by catalyzing the formation of OH^−^ radicals from the non-enzymatic chemical reactions between superoxide and H_2_O_2_ [211]. Alternatively, the positive effect of CuONPs at an optimum concentration on callus induction can be explained as Cu being an essential nutrient in plant growth and acting as a co-factor in many metalloproteins. Cu also acts as a structural element in regulatory proteins and is involved in important physiological processes like the electron transport chain, hormone signaling, and cell wall metabolism [212]. Studies revealed that the application of CuONPs counteracts stress by changing various biochemical reactions in *V. radiate* [71], *C. arietinum* [66], and *B. nigra* [5] (Table 2). Ti is another essential element that also increases the nutrient absorption of metals like Ca, Mg, Zn, and P [213]. It has been reported that TiO_2_NPs at a proper concentration promotes plant growth by assisting water absorption inplant cells and inducing cellular metabolism [214], by activating photosynthetic complexes, Rubisco carboxylase activity and nitrogen metabolism in the plant cell [215,216]. Mandeh et al. [42] reported that TiO_2_NPs facilitated plant growth as they play a role similar to PGRs like cytokinin and gibberellic acid (GA_3_), whereas SiO_2_NPs had increased the levels of GA_3_ in the cells, having a plant hormone-like property and play a vital role in cell division, and consequently increased the elongation [217]. TiO_2_NPs showed an influence on cultures of *L. usitatissimum* cv. Kerman Shahdad for variation in enzyme activities [82]. Gowayed et al. [94] studied *S. tuberosum* cv. Sante and Proventa under SiO_2_NPs influence and observed that it increased the number of protein bands in both cultivars compared to control and NaCl treatment. This increase in bands indicated that SiO_2_NPs activated genes which are important proteins associated with salt-stress resistance. Elevated activities of antioxidant enzymes (GPX and SOD) were also observed (Table 2).

The role of ethylene in in vitro plant regeneration has been well documented [218], and Ag^+^ ions are known to inhibit ethylene action by replacing Cu^+2^ ions with Ag^+^ and blocking ethylene receptor (ETR1) [219]. The beneficial effect of Ag can also be attributed to enhance polyamine biosynthesis rather than reduce ethylene production [220], and an increase in auxin efflux independent of ethylene response that affects plant growth [221]. The supplementation of AgNPs can enhance the plant cell’s nutrient and water uptake from culture media by mutilating the cell wall [222]. Another hypothesis suggested that AgNPs modify the structural components of cellular membranes, and macromolecules, influence cell division and defense systems, and interfere with the physiological and biochemical processes of plants by altering the gene expression [223]. But, the higher concentration of AgNPs cause lipid peroxidation because of the ROS generation, inhibition of ethylene production, and restriction in the electron transport chain of mitochondria and chloroplast, which all lead to oxidative burst, rise in ROS concentration, and eventual cell death [200,224]. Whereas at low concentrations, it modulates the redox status of plants, because of its efficient catalytic activity in redox reactions by acting as electron relay centers [225] and its ability to support electron exchange with Fe^2+^ and Co^3+^ [226]. Vannini et al. [227] observed that AgNPs cause changes in proteins involved in redox regulation and sulfur metabolism; they also alter some proteins related to the endoplasmic reticulum and vacuole. In *B. juncea* var. pusa jaikisan, they have increased chlorophyll content and the activities of enzymes, but MDA, proline, and H_2_O_2_ content were decreased [189]. Similarly, decreased MDA, proline, and H_2_O_2_ levels were also observed after AgNP treatment in cultures of *O. sativa* cv. IR64 [88]. In addition, variation in response has been well documented between crop species, as in some crops the contents were found to be decreased, e.g., *B. oleracea* var. *sabellica* ‘Nero di Toscana’ [59]. On the contrary, increased metabolites and enzyme activities have been reported in *M. charantia* [84], *Caralluma tuberculata* N.E.Br. [76], and *Maerua oblongifolia* (Forssk.) A. Rich [83]. Jamshidi et al. [78] had observed that AgNPs in the cell suspension culture of *C. avellana* cv. Gerd Eshkevar showed a positive influence on the contents of ascorbate peroxidase (APX), CAT, H_2_O_2_, and PAL, but they decreased SOD and POD activities, and total soluble phenol content. Recently, in *B. napus* shoots, they increased metabolites and H_2_O_2_ levels but no change in phenolics was found [60]. The examples of different NPs on biochemical changes in seedlings and different cultures of important crops are given in Table 2.

NPs after internalization evoked changes at molecular levels as metal NPs are known to induce systemic stress, and to overcome this stress, they alter the expression of genes [228]. CNT treatment is known to affect the expression of the water channel proteins (aquaporins) [229] that are considered to be crucial for the process of seed germination and plant growth [230]. The expression of water channel genes (aquaporin, *LeAqp2*) is reported to be activated in response to MWCNTs in tomato seedlings [73,229] and tobacco cells [231]. Villagarcia et al. [73] revealed that MWCNTs affect the expression of genes regulating cell division and cell wall extension in treated cells, resulting in faster growth than the unexposed control cells. However, a few in vitro studies that were carried out on gene expression analysis, e.g., Nair and Chung [66] analyzed the effect of CuONPs on seedlings of *C. arietinum* and correlated the biochemical changes with oxidative stress response genes such as *SOD* and *CAT*, but there was no significant change in the expression of *APX*. Likewise, on seedlings of *B. rapa* ssp. *rapa*, AgNPs up-regulated the expression of different genes related to antioxidant defense (catalase, *CAT*; peroxidase, *POD*; glutathione S-transferase, *GST*), biotic and abiotic stresses (pathogenesis-related gene 1, *PR1*; lipoxygenase 2, *LOX2*), carotenoids (β-cyclase, *CYB*; zeaxanthinepoxidase-1, *ZEP1*), anthocyanins (production of anthocyanin pigment 1, *PAP1*; anthocyanin synthase, *ANS*; phenylalanine ammonia-lyase, *PAL*), and glucosinolates (*BrMYB28*; *BrMYB29*; *BrMYB34*; *BrMYB51*; sulfotransferase, *St5C*; and superroot1, *SUR1*). Further, over-expression of the *Geranyl diphosphate synthase* gene (*GPPS* gene), a key gene involved in the thymoquinonebiosynthesis pathway, has been observed in *N. sativa* after TiO_2_ and SiO_2_NPs [232]. Manickavasagam et al. [88] depicted AgNPs in media containing *O. sativa* L. cv. IR64 seeds showed up-regulation of ethylene (*ERF063*), ABA (*OsRab16*), auxin (*OslAA1*), cytokinin (*RR2*), and gibberellic acid (*PBZ1*) responsive genes, justifying the stress induced by NPs treatment. In addition, treatment of *M. charantia* with selenium nanoparticles (SeNPs) showed a variation in methylation-susceptible loci (MSL) between the control and treated group which suggested an epigenetic modification in response to NPs. The results also revealed that there was significant up-regulation of transcription factor *WRKY1*, and genes like *PAL* and 4-coumarate:CoA ligase (*4CL*) [233].

## 6. NPs as a Tool for Genetic Engineering in Crops

Genetic engineering has proven useful in the face of climate change and the growing global population by bestowing desirable genetic traits and enhancing crop productivity. The delivery of genetic materials such as DNA and small interfering RNA (Si-RNA) is important for the development of pest, pathogen, and stress-resistant strains of crops by altering the gene expression [234,235]. The bottleneck in genetic transformation is the plant cell wall, which causes obstacles such as targeting the delivery system, transportation through the cell membrane, uptake and degradation in endolysosomes, and intracellular trafficking of DNA to the nucleus [236]. Similarly, the traditional delivery methods also have some demerits like viral gene vectors have a narrow host range, allowing only a limited size of genetic material to be delivered, and they also face the possibility of inducing viral symptoms. Other methods are microinjection, *Agrobacterium*-mediated transformation, and microprojectile bombardment. All these methods had either very low efficiency (0.01–20%) or were mainly applied for dicotyledons [11]. Another concern behind *Agrobacterium*-mediated transformation is the usage of antibiotics such as carbenicillin, cefotaxime, rifampicin, and timentin for the removal of bacteria after co-cultivation, which affected the regeneration potential and genetic stability of the regenerated plantlets [15]. An alternative method for genetic engineering like genome editing using CRISPR/Cas9 is comparatively precise and can manipulate the genome, but it also relies on an *Agrobacterium*-mediated pathway and thus has drawbacks like undesirable off-target effects and insertional mutations in the genome [236]. To circumvent these obstacles, a technique that enables specific horizontal gene transfer is required that allows the delivery of genes into abroad range of plant species without the need of external force to induce desirable traits in commercially important crops [237].

NPs having an extremely small size and easy uptake into plant cells are a potential vehicle for passive gene transfer in different tissues like seeds, leaves, calli, roots, etc. [8,238]. The charge and shape of NP greatly influence the cell membrane translocation, and thus these properties are central to nanocarrier optimization. Another benefit of NP-mediated delivery is that it has high DNA-binding ability and thus has high transformation efficiency without genome integration [239,240]. It has been commonly reported that the internalization is faster and more efficient for cationic NPs as they easily bind with negatively charged cell membranes in comparison to anionic NPs [241]. The conjugation of DNA with NPs and transformation in the cell cytoplasm integrates DNA into the target genome, and develops the transgenic plants with desired traits [242]. Nonetheless, fewer reports are available on the usage of nanomaterials as carriers to deliver biomolecules into the in vitro cultures of crops as compared to research available on morphogenesis. The first study on gene transfer using NP has been documented by Torney et al. [243] where gold-capped mesoporous silica nanoparticles (MSNs) were delivered to *N. tabacum*. Liu et al. [244] synthesized starch NPs and coated them with poly-*L*-lysine and fluorescent material Ru(bpy)_3_^2+^·6H_2_O. To deliver the DNA, they conjugated NPs with pEGAD plasmid DNA and successfully transformed the suspension culture of *Dioscrea zigiberensis* G H Wright. Furthermore, poly-*L*-lysine-coated ZnS nanoparticles with an average size of 3–5 nm efficiently delivered the β-glucuronidase (*GUS*)-encoding plasmid into young tobacco leaves using the ultrasonic treatment. The efficiency of gene transfection of the treated tobacco plant under various conditions indicated that the highest efficiency is achieved when an ultrasonic treatment with intensity of 60 W for 20 min is applied. These results indicated that the optimum condition for the ultrasonic treatment to achieve the highest gene transfection efficiency depends on the plant type (protoplast, cells, leaves, roots, etc.) as well as nanocarriers and their size [245].

The carbon-based NPs have also been proven as an efficient system; Vijayakumar et al. [246] found that the carbon-supported AuNPs delivered *GUS* genes more efficiently as compared to the gold particles using a gene gun into *N. tabacum*, *O. sativa* and *Leucaena leucocephala* (Lam.) de Wit. Similarly, positive results have been obtained for fluorescein isothiocyanate (FITC)-tagged SWCNTs and complexes of FITC-tagged DNA molecules with MWCNTs in the suspension cultures of *N. tabacum* BY-2 cells [172]. In addition, the genetic transformation of *N. tabacum* protoplasts with a plasmid construct pGreen 0029 having a *yfp* reporter was carried out using SWCNTs and MWCNTs. It was found that SWCNTs were able to transform both protoplasts and walled plant cells, whereas MWCNTs could only transform the protoplasts because of the presence of a cellulose wall which hindered NP penetration [247]. Later on, FITC has been delivered into *B. napus* var. Jet Neuf and *D. carota* var. Konservnaja 63 protoplast using SWCNTs [248] and magnetic AuNPs [249], suggesting successful delivery of the molecules.

An interesting study was carried out to transfer pCambia 1301 having the *GUS* gene into *B. juncea* cv. pusa jaikisan, where a better transformation efficiency was achieved with calcium phosphate nanoparticles (CaPNPs, 80.7%), followed by *A. tumefaciens* (54.4%) and naked DNA (8%) [250]. Similarly, the utilization of CaPNPs to deliver the pBI121-harboring GFP gene into tobacco cells was reported by Ardekani et al. [251]. In corroboration with these, usage of CaPNPs in *C. intybus* to deliver the *HMGR* gene showed a positive transformation as higher chlorophyll, proteins, and esculin contents, as well as higher HMGR activity, were detected [252]. For the efficient and stable transformation of *Jatropha curcas* L. callus [253] and cell suspension [254], a complex of CdSe fluorescent quantum dots (QDs) with *L*-cysteine and chitosan–DNA (CS-DNA) NP conjugate have been reported. In many reports, MSNs have been documented as an efficient nanocarrier; e.g., Martin-Ortigosa et al. [255] documented the usage of MSNs in different ways like gold plating for MSNs, CaCl_2_/spermidine DNA coating, and NPs with gold microparticles and gold nanorods to enhance the NP-mediated DNA delivery using the biolistic method in onion, maize, and tobacco. In another study on the same plants, protein-loaded Au-MSNs can be subsequently coated with plasmid DNA and introduced into plant tissues through particle bombardment by which both protein and DNA can be transferred efficiently [256]. For instance, MSNs have been well documented as a carrier to deliver Cre recombinase protein into the *Z. mays* [257], *cryIAb* gene in *S. lycopersicum* var. falat [258], and *GUS* gene in *N. tabacum* [259].

Furthermore, layered double hydroxide (LDH) nanosheets have shown positive results in transforming *N. tabacum* with fluorescent dyes such as tetramethyl rhodamine isothiocyanate (TRITC), FITC, and DNA molecules [260]. New polymeric dimethylaminoethyl methacrylate (DMAEM)-based polymer NPs have been reported to carry the *yfp* gene in *N. tabacum* protoplast with the help of polyethylene glycol (PEG) [261]. Similarly, Zhao et al. [262] used magnetic Fe_3_O_4_NPs and documented the stable transformation of the *BTΔα-CPTI* gene in *Gossypium hirsutum* Linn. pollen, which remained integrated into the genome, which transcribed, expressed and produced an insect-resistant transgenic progeny of cotton plants. Later on, *S. tuberosum* (cv. lady and spunta)-resistant varieties were generated for pathogenic fungi like *Alternaria alternate* and *Rhizoctonia solani*, with the help of two thionin genes delivered using NPs [263]. Gil-Humanes et al. [264] also successfully transformed Wheat dwarf virus (WDV)-derived replicons along with CRISPR/Cas9 to induce targeted mutagenesis in *T. aestivum* cv. Bobwhite. Recently, green synthesized FeNPs using the leaf extract of *Camellia sinensis* were utilized for the successful and stable transformation of pBIN.35s-mgfp5-ER carrying *GFP* gene to the *Abelmoschus esculentus* [265] and *UidA* gene with the help of chitosan NPs to the in vitro plants of *S. tuberosum* [266].

## 7. Conclusions and Future Perspectives

The world population is expected to reach 9.6 billion by 2050 and to feed this ever-increasing population, there will be increased pressure on land, which is not extendable. A higher usage of fertilizers causes soil damage and environmental pollution. Recent advancements in the field of nanotechnology have demonstrated the potential to revolutionize agricultural production. However, the concern raised behind the in vivo usage of NPs is their release in the environment which might result in the accumulation of different NPs in each trophic level of the ecological pyramid; therefore, we should use NPs judicially.

An alternative strategy to test the NPs is to use them in a tissue culture system which is a powerful tool for the screening of plantlets; it also provides a unique opportunity for studying many aspects of plant growth and development under well-defined and controlled environmental conditions. NPs have been shown to enhance plant germination/production, improve plant resistance to abiotic and biotic stress, assist efficient nutrient exploitation, and promote plant growth, with reduced environmental impact compared to traditional approaches with bulk material. The main application of NPs under in vitro conditions is to increase the crop yield, and ability of plantlets to cope with stressful conditions by which it will adapt to the conditions before transplanting into the field. However, the reports reviewed in the present manuscript suggested that the type and concentration of NPs, as well as the crop species and even cultivar, showed variation in responses. Therefore, the effect of different types and concentration ranges of NPs on plant tissue should be optimized accordingly to determine the optimum dose, which usually ranges between 1–50 mg/L. This optimization will be useful in precision agriculture for individual crops. Further, the usage of NPs as an elicitor has been explored which showed promising results for many commercially valuable metabolites; hence, nano-elicitors may be exploited for the commercial production of secondary metabolites at the bioreactor level.

Although many reports are available on NPs’ effect on plants under in vivo conditions, to gain a clear understanding of the underlying mechanisms behind the role of NPs in plant morphogenesis, these can be further astudied via in vitro routes. The advantage of NPs is that they are promising materials for biomolecule delivery, owing to their ability to traverse plant cells without external force, their tunability for diverse cargo conjugation, and broad host range applicability. These qualities make them a promising tool for the genetic engineering of plants for an easier delivery of genes and without injury to the plant cells. They can also be used for targeted gene delivery to the nucleus, chloroplast, and mitochondria to achieve transgenesis in plants. Reports on molecular mechanisms of elicitation using NPs are scant; hence, systematic omics-based analyses (e.g., genomics, transcriptomics, proteomics, and metabolomics) are necessary.

## Figures and Tables

**Table 1:** Effects of various NP on seed germination ofdifferent crops under in vitro conditions.

Plant	Nanoparticle (NP) Treatment	Parameters	Reference
*Brassica juncea* var. pusa jaikisan	AgNPs	Enhancement in the growth of seedlings in terms of shoot FW, shoot and root length, and vigor index	[62]
*Brassica napus*	AgNPs/AuNPs	Deceased shoot and root lengths, as well as shoot FW and DW	[60]
*Brassica nigra*	ZnONPs	Increased shoot length and shoot DW, decreased root length, shoot FW, root FW and DW	[65]
*Brassica nigra*	CuONPs	Delayed seed germination, decreased plantlet length, and their FWs and DWs	[5]
*Brassica oleracea* var. *sabellica* ‘Nero di Toscana’	AgNPs	Increased germination response, shoot and root lengths, as well as biomass	[59]
*Cicer arietinum*	CuONPs	Decreased shoot and root lengths, FWs and DWs of shoot and root, increased lignifications in root cells	[66]
*Eruca sativa*	AuNPs, CuNPs and AgNPs	AgNP-increased seed germination, shoot and root lengths, and seed vigourindex; AuNP-and CuNP-decreased seed germination, shoot and root lengths, and seed vigour index	[56]
*Glycine max* hybrid S42-T4	MWCNTs	Early and better germination, increased shoot, root and leaf lengths, shoot and root FWs and DWs	[67]
*Hylocereus undatus*	AgNPs	Increased germination, shoot number, shoot, and root lengths, cladode size, and FW	[63]
*Hordeum vulgare* hybrid Robust	MWCNTs	Early and better germination, increased shoot, root, and leaf lengths, shoot and root FWs and DWs	[67]
*Linum usitatissimum* cv. Barbara	ZnONPs	Increased shoot and root length, as well as their FWs and DWs	[68]
*Nicotiana tabacum*	AgNPs	Increased germination and dry biomass	[61]
*Oryza sativa* cv. Swarna	AgNPs	Increased shoot and root length, FWs and DWs of shoot and root	[57]
*Pennisetum glaucum*	AgNPs	Increased germination, seed vigour index, shoot and root lengths, and fresh and dry biomass	[58]
*Petroselinum* *crispum*	TiO_2_NPs	Increased germination, shoot and root lengths, and their FWs	[69]
*Phaseolus radiatus*	AgNPs	Adverse effect on seedling growth	[37]
*Phaseolus vulgaris*	AgNPs	Increased seed germination, shoot and root length, their FWs and DWs, number of axillary buds, adventitious buds and leaves	[64]
*Physalis peruviana*	AgNPs	Decreased shoot and root lengths, chlorophyll content, but increased FW and DW	[70]
*Raphanus sativus* var. sativus ‘Ramona’	AgNPs	Increased germination response, shoot and root lengths, and seedling biomass	[59]
*Solanum lycopersicum* var. Poranek	AgNPs	Decreased germination response, shoot and root lengths, and seedling biomass	[59]
*Sorghum bicolour*	AgNPs	Adverse effect on seedling growth	[37]
*Vigna radiata*	CuONPs	Decreased shoot and root lengths and their FWs, increased lignifications in root cells	[71]
*Zea mays* hybrid N79Z 300GT	MWCNTs	Early and better germination, increased shoot, root and leaf lengths, shoot and root FW and DW	[67]

AgNPs: silver nanoparticles; AuNPs: gold nanoparticles; CuNPs: copper nanoparticles; CuONPs: copper oxide nanoparticles; DW: dry weight; FW: fresh weight; MWCNTs: multi-walled carbon nanotubes; TiO_2_NPs: titanium dioxide nanoparticles; ZnONPs: zinc oxide nanoparticles.

## Data Availability

Not applicable.
